# Tobacco consumption among ninth-grade students in Chisinau, Moldova

**DOI:** 10.18332/tid/142105

**Published:** 2021-10-01

**Authors:** Aculina Topadă, Valentin Nădăşan, Monica Tarcea, Zoltán Ábrám

**Affiliations:** 1 Department of Hygiene, George Emil Palade University of Medicine, Pharmacy, Science, and Technology of Targu Mures, Targu Mures, Romania; 2 Department of Community Nutrition and Food Safety, George Emil Palade University of Medicine, Pharmacy, Science, and Technology of Targu Mures, Targu Mures, Romania

**Keywords:** smoking, adolescents, Moldova

## Abstract

**INTRODUCTION:**

The aim of this study was to evaluate the potential impact of the changes in the national legislation on tobacco consumption and the impact of the implementation of the National Tobacco Control Program on ninth-grade students in Chisinau, Moldova, during 2015 and 2018, as well as tobacco consumption behavior and exposure to pro- and anti-tobacco messages.

**METHODS:**

The study sample consisted of the ninth-grade students from 132 schools in Chisinau, Moldova. It included 20 randomly selected schools (3 gymnasiums and 17 high schools). Data were collected using a validated self-completed questionnaire, once in October–December 2015 (n=368) and repeated in February–April 2018 (n=819). The chi-squared test was applied to compare categorical variables between the 2015 and 2018 groups.

**RESULTS:**

The student participants had mean age of 14.8 ± 0.49 years (2015) and 14.9 ± 0.53 years (2018), and, by coincidence, both groups had 51.9% boys and 48.1% girls. However, despite the rather strict regulations on tobacco in Moldova, adolescents still report a significant increase in the prevalence of cigarette consumption in the last 30 days in the period from 2015 to 2018. In this period, anti-smoking messages increased significantly and were seen by students in shopping malls, supermarkets (35.1% in 2015 and 60.0% in 2018), cinemas (11.7% in 2015 and 36.5% in 2018), magazines and newspapers (19.3% in 2015 and 37.6% in 2018) (p<0.001).

**CONCLUSIONS:**

Tobacco consumption among ninth-grade pupils had increased between 2015 and 2018 in Chisinau, Moldova. Adolescents represent a critical subpopulation of smokers and tobacco control experts must concentrate their efforts to reduce their exposure to pro-smoking messages and increase their exposure to anti-smoking messages (via internet, sport events, etc.).

## INTRODUCTION

Tobacco consumption represents a significant public health problem in the Republic of Moldova and the World Health Organization (WHO) estimated that in 2010 approximately 23% (690200 people) of its population were smokers^1^. In 2009, significant efforts to combat tobacco consumption by acceding to the World Health Organization Framework Convention on Tobacco Control were made, and in 2012 the first National Program on tobacco control for the years 2012–2016 was approved^2^. In September 2015, the first amendment to the law on tobacco products was approved^3^.

In 2011, the prevalence rate of smoking in the adult population (18–69 years) was 25% with a slight decrease in 2013 to 23.3%^4,5^. The rate of smoking continued to decrease to 21% in 2014 but increased in 2017 to 25%^6,7^.

Recent studies have shown that the prevalence of tobacco smoking among adolescents aged 15 years is different among cities in Europe. The average prevalence rate of daily smoking was: 26.8% in Latina (Italy), 24.5% in Namur (Belgium), 16.8% in Tampere (Finland), 12.2% in Hannover (Germany), 12.4% in Amersfoort (Netherlands), and 19.7% in Coimbra (Portugal)^8^. Other studies also demonstrated that the socioeconomic status of school and family influence the smoking behavior of adolescents. The prevalence of daily smoking was higher in schools with low socioeconomic status and among students from families where the parents had a low education level^9^.

The European School Survey Project on Alcohol and Other Drugs study (ESPAD) found the following lifetime smoking prevalence rates among adolescents aged 13 years from Europe: 66% Czech Republic, 65% Lithuania, 62% Croatia and Slovakia, 58% Romania, and 33% Moldova. The highest prevalence rate of students who smoked cigarettes during the last 30 days was found in Italy (37%), Romania (30%), and Moldova (9%). This study found the most significant gender differences in the lifetime use of cigarettes in Moldova (50% for boys vs 15% for girls)^10^. In Europe, the ESPAD study^11^ in 2019 found on average that 20% of students had smoked cigarettes during the last 30 days and 41% of students ever smoked cigarettes. The same study reported the following prevalence rates among the students who smoked cigarettes during the last 30 days: 21% Spain, 22% Ukraine, and 31% Romania. According to the Leal-López study^12^, the daily smoking prevalence among Spanish adolescents aged 15–18 years in 2018 was 8.7%, and the prevalence among adolescents aged 15–16 years who smoked less than once a week was approximately 6%.

The most recent survey, in Moldova in 2016 involving young people aged 14–29 years, revealed that the daily tobacco use prevalence was 16.5% and for occasional use 7.2%. About 13.7% of young men aged 14–19 years smoked daily and 12.4% occasionally, while the prevalence rate among young women of the same age group was only 1.2% for daily and 1.7% for occasional smoking^13^. A study conducted among adolescents in Moldova in 2015 showed that about 30% of students in the 8th and 9th grades smoked at least once during their lifetime. This study included students from rural and urban areas, and showed that lifetime smoking prevalence among students in large urban centers was higher (38.5%) compared to other places of residence (30.2% urban; 27.9% rural)^14^.

The study^15^ conducted in 2016 analyzed risky behaviors among adolescents in Moldova, and applying only a few questions about tobacco use found that 9.9% of students aged 15 years were current smokers in 2014.

The prevalence of tobacco consumption in Romania among the ninth-grade students was estimated at 48.4% for those who have tried to smoke at least once, and 21.4% for those who were current smokers^16^. Among resident children from foster-care homes in four Transylvanian counties, 44.6% had tried to smoke cigarettes at least once and about one in four (25.9%) reported that they smoked cigarettes in the past 30 days^17^. Another study among institutionalized children in family-care homes in Romania found that 61% of the children from Harghita county and 54.3% from Mures county tried to smoke cigarettes^18^.

Assessment across the 27 European Union (EU) Member States showed that from 2006 to 2014, the prevalence rate of smoking had decreased in Europe. The higher implementation level of tobacco control policies is associated with a lower prevalence rate of smoking, but the decrease of the prevalence rate varies across the countries. In some countries, the prevalence rate has fallen by more than 40% (Sweden, Latvia) while in others it has fallen insignificantly or not at all, as in Bulgaria and France^19^.

The aim of the present study was to evaluate the impact of the national legislation on tobacco consumption and also of the National Tobacco Control Program upon the ninth-grade students in Chisinau, Moldova, during the period 2015–2018, as well as tobacco consumption behavior and exposure to pro- and anti-tobacco messages.

## METHODS

### Sample and data collection

The study sample consisted of 9th grade students from 132 schools located in Chisinau, the capital of the Republic of Moldova. It included 20 randomly selected schools (3 gymnasiums and 17 high schools). Data were collected using a self-completed questionnaire, once in October–December 2015 (368 respondents) and repeated in February–April 2018 (819 respondents). The inclusion criteria were schools: 1) having at least one ninth-grade class; 2) teaching in Romanian; 3) located in Chisinau, but not in the suburbs; 4) with no special sports or health profile; 5) with no evening classrooms (where students are older than 18 years); 6) which had an IT class; and 7) where it was possible to obtain the agreement of the school director to participate in the study. The list of eligible schools in the municipality was obtained from the General Directorate of Education, Youth and Sports (DGETS) of the Chisinau Municipal Council. After analyzing all the schools, 41 high schools and 7 gymnasiums qualified. The study sample was chosen randomly and consisted of 17 high schools and 3 gymnasiums, in total 20 pre-university educational institutions. The sample size was calculated using a 95% confidence level and an expected proportion of 50% from an estimated population size of 3500 pupils from 48 schools. Also, we doubled the number of subjects in order to adjust for the cluster effect.

The survey was conducted in two stages; during the first stage, implemented in the period October–December 2015, the questionnaire included 368 students; in the second stage, which was implemented in the period February–April 2018, the same educational institutions were included, except for one high school that did not have a ninth grade in the period 2017–2018.

The first group in 2015 completed the online questionnaire in the computer class. The second group in 2018 completed an onsite paper-andpen questionnaire. Fifteen classes from one school declined to participate due to organizational reasons. The remaining sample included 19 schools, 40 classes; therefore, out of 1531 students enrolled in the ninth grade, 819 students were interviewed. In 237 cases, the students refused to fill in the questionnaire; in 475 cases, their parents refused to accept their participation in the study or did not submit their written consent before completing the questionnaire.

The study obtained the approval of the Ethics Commission of Scientific Research of the University of Medicine, Pharmacy, Sciences and Technology of Targu Mures, Romania, where the ASPIRA program was implemented. Parents were informed of the purpose, benefits and risks of the research and gave their written consent before conducting the questionnaire. The written consent consisted of two parts: in the first was included general information about the questionnaire, explaining what the questionnaire consists of, by whom it is carried out and what data were required from the students; in the second they were required to enter personal data and confirm that they agreed with the use and processing of the personal data in the study, according to the national legislation in force.

The validated questionnaire applied in Chisinau was taken from the project ‘The school Action Software to Prevent Interactive Smoking in Romania (ASPIRA)’ implemented within the program Building Capacity for Tobacco Research in Romania: A Partnership among Romanian, American, and Hungarian Scientists. The questionnaire used for the study was that from the ASPIRA program applied in Targu Mureş, Romania, in 2016^16^. The questionnaire was structured in such a way as to collect general data about students, followed by data related to the smoking behavior of students and their family members. It included questions about the motivation to smoke or not, the temptation to smoke or the confidence to resist the temptation.

### Measurements

Smoking status was assessed by collecting information about ever trying conventional cigarettes and past 30-day use of conventional cigarettes. The following sociodemographic data were collected: age, gender, and ethnicity. Social influences were measured by perceived household members’ and peers’ smoking status (smoking status of the father, mother, siblings, close friends). Two items measured the variables for exposure to pro- and anti-smoking messages: ‘Please choose the types of pro-smoking messages that you have seen or heard in the last month’ (yes/no) and ‘Please choose the types of anti-smoking messages that you have seen or heard in the last month’ (yes/no). The questions investigating exposure to pro- and anti-smoking messages covered the most common sources such as radio, television, outdoor advertising, cinema, newspapers, internet, sports events, shops, and supermarkets. The variables are described in detail in Nădăşan et al.^20^.

### Statistical analysis

Means and standard deviations were calculated for the age of the participants. Absolute and relative frequencies were calculated for all the categorical variables. Chi-squared test was applied to compare categorical variables from 2015 with those from 2018. The threshold for statistically significant differences was set at 0.05.

## RESULTS

Table 1 presents the sociodemographic variables of the investigated samples. The 2015 sample had a mean age of 14.8 ± 0.49 years, with a slightly higher average age of 14.9 ± 0.53 years in 2018. The respondents were 51.9% boys and 48.1% girls, for both study groups.

**Table 1:** Sociodemographic characteristics of the ninth-grade students, Chisinau, Moldova, 2015 and 2018

*Characteristics*	*2015 (n=368) n (%)*	*2018 (n=819) n (%)*	*p*
**Age** (years), mean ± SD	14.8 ± 0.49	14.9 ± 0.53	0.02*
**Sex**			
Boys	191 (51.9)	425 (51.9)	0.998**
Girls	177 (48.1)	394 (48.1)	
**Ethnicity**			
Moldovan (Romanian)	353 (95.9)	779 (95.1)	0.540***
Ukrainian	3 (0.8)	4 (0.5)	
Russian	5 (1.4)	20 (2.4)	
Other	7 (1.9)	16 (2.0)	

* Value of p was calculated using the t-test.

** Chi-squared test, two-tailed p.

*** Chisquared test compared the Moldovan ethnics with all the other ethnics combined.

Table 2 presents the characteristics of the smoking status of the respondents for 2015, and for 2018 which was used as a post-intervention group. Smoking prevalence and intensity were significantly higher in 2018; about 55.8% of students ever tried cigarettes, and 46.9% of students used cigarettes in the last 30 days. Having friends, parents or siblings who smoked was also more common in 2018.

**Table 2:** Smoking status of ninth-grade students, their parents, siblings and friends, Chisinau, Moldova, 2015 and 2018

*Variables*	*2015 (n=368) n (%)*	*2018 (n=819) n (%)*	p*
**Smoking status of students**			
Ever tried cigarettes	166 (45.1)	457 (55.8)	0.001
Last 30 days cigarette users	55 (14.9)	384 (46.9)	<0.0001
**Smoking status of parents, siblings and friends**			
Father smoker	103 (28.0)	261 (31.9)	0.180
Mother smoker	25 (6.8)	96 (11.7)	0.009
Brother/sister smoker	49 (13.3)	155 (18.9)	0.017
Close friends smokers	182 (49.5)	478 (58.4)	0.004

* Chi-squared test, two-tailed p.

Details about the exposure of the ninth-grade students to pro- and anti-smoking messages are presented in Table 3. The study shows that the most effective and stable ways to reach students with antismoking messages is via radio/television and the internet. These sources have a rate of >50% both until the implementation of the legislation requiring the publication of such messages and after its implementation. Approximately 60% of respondents accepted that they saw or heard advertisements with anti-smoking messages in commercial spaces in 2018, compared to 35.1% of students in 2015. The sources of exposure that increased due to the implementation of the legislation were: cinema from 11.7 % (2015) to 36.5% (2018); and newspapers/magazines from 19.3% (2015) to 37.6% (2018). In contrast, the source of lowest exposure (26.9%), which did not increase significantly (30.6%) with the implementation of the legislation, were sports events.

**Table 3:** Exposure of ninth-grade students to prosmoking and anti-smoking messages, Chisinau, Moldova, 2015 and 2018

*Source of exposure*	*2015 (n=368) n (%)*	*2018 (n=819) n (%)*	p*
**Pro-smoking messages**			
Radio/television	67 (18.2)	162 (19.7)	0.525
Outdoor advertisement	49 (13.3)	112 (13.7)	0.867
Shopping areas	37 (10.1)	10.1	0.966
Cinema	14 (3.8)	28 (3.4)	0.739
Newspapers/magazines	18 (4.9)	36 (4.4)	0.705
Internet	106 (28.8)	232 (28.3)	0.866
Sport events	24 (6.5)	53 (6.5)	0.974
Other	37 (10.1)	90 (11.0)	0.630
**Anti-smoking messages**			
Radio/television	204 (55.4)	489 (59.7)	0.167
Outdoor advertisement	180 (48.9)	393 (48.0)	0.767
Shopping areas	129 (35.1)	491 (60.0)	<0.001
Cinema	43 (11.7)	299 (36.5)	<0.001
Newspapers/magazines	71 (19.3)	308 (37.6)	<0.001
Internet	207 (56.3)	464 (56.7)	0.897
Sport events	99 (26.9)	251 (30.6)	0.191
Other	80 (21.7)	187 (22.8)	0.676

* Chi-squared test, two-tailed p.

Although Article 21 of the law which entered into force on 1 January 2018, which prohibits any form, direct or indirect, of advertising in favor of tobacco products, there has been no significant decrease in any of the sources of exposure to the messages of promotion of tobacco products. The most used source to which the students claimed to have been exposed was the internet in 2015 (28.8%) and in 2018 (28.3%). Just under 20% saw or heard pro-smoking messages through television and radio in both groups.

## DISCUSSION

The public health problem related to adolescent smoking in Moldova is significant. However, the legislative changes and the implementation of European programs began much later than in some other European countries. A large part of the proposed measures entered into force gradually or remained not fully implemented. The political/economic situation of the Republic of Moldova and the signing of its Association Agreement with the European Union make it possible to implement new programs and action plans to reduce the prevalence rate of smoking among adolescents.

In this study were reported similar rates of current smoking compared with the general population of adolescents in Moldova in 2015 (13.7% vs 14.9%, respectively); in 2018 the current smoking was seven times higher than the prevalence reported in the GYTS (7.4% vs 46.9%, respectively). These prevalence rates are comparable with previous research in Moldavian students^21-24^. Ever trying cigarettes (45.1% in 2015) was also similar to previous findings for the GYTS^21-24^, while for the sample in 2018, we found a markedly higher prevalence of 55.8% (p<0.001).

Exposure to tobacco promotion is associated with the likelihood of teens starting to smoke^25^. Based on this association, we decided to study the level of implementation of tobacco control advertising in Moldova. Although, since 2003, the World Health Organization under Article 13 of the Framework Convention on Tobacco Control has laid down a comprehensive ban on tobacco advertising, promotion and sponsorship, Moldova only ratified this Article in 2015. Until the ratification, some studies^21-23^ on adolescents in Moldova gave a proportion of >45% of students who saw pro-smoking messages at a shopping area, and >75% from radio/television. In our study, the exposure to the pro-smoking messages was 3 times lower than the studies conducted on Moldovan students^21-23^, especially those in the media and commercial spaces.

Exposure to the anti-smoking messages in our study had a significantly higher prevalence in 2018 than in 2015, especially the media exposure sources. Studies of adolescents have shown that anti-smoking media campaigns can help to reduce the prevalence of smoking by discouraging young people from starting smoking and by encouraging current smokers to quit^26^.

The originality and timeliness of the study come through the comparison of data on tobacco use among students after the approval of the law on tobacco use in the Republic of Moldova. These surveys provide data on the correlation between the students’ family and school and their influence on student behavior regarding smoking. Also, the study makes for the first time a comparison of the changes that appeared in the frequency of exposure to advertisements by the students at the beginning of the implementation of the law and after two years.

### Legislation implemented

During the period 2015–2018, when the survey was conducted, in Moldova were approved and entered into force several amendments to the law on tobacco consumption. These amendments were proposed and approved as a result of Moldova’s ratification of the WHO Framework Convention on Tobacco Control. The latest modification was adopted on 29 May 2015, and entered into force on 17 September 2015. The particularity of this law is that each amendment of the law enters into force on different dates. The most relevant amendment for our study was Article 21 on advertising and sponsorship of tobacco products by banning the advertising and promotion of tobacco products, which entered into force on 1 January 2016^3^.

Based on this law, the National Program on Tobacco Control for 2017–2021 was developed and implemented. The goal of the program was to improve population health through the reduction of tobacco consumption and the full implementation of the WHO Framework Convention on Tobacco Control^27^. Also in Moldova, other regulations on tobacco consumption entered into force, such as Government Decision No. 1065 of 19 September 2016 for the approval of the sanitary regulations regarding tobacco products and related products, and Government Decision No. 613 of 1 August 2017 for the approval of the sanitary regulation on health warnings and labeling of tobacco products, tobacco intended for the rolling of cigarettes and related products^28,29^.

The National Program on Tobacco Control for 2017–2021 and the Action Plan for its implementation imposed strict rules for the media, recommending to disseminate aggressive content with anti-smoking messages. The program also provides recommendations for the organization of information campaigns for promoting anti-smoking messages by the municipal councils of the town halls at the local level. Thus, several anti-smoking messages appeared in public spaces, supermarkets, and cinemas^27,28^.

The Inspectorate of the National Agency for Public Health was responsible for the monitoring the implementation of the law on prohibition of pro-smoking messages; taking into consideration that the Agency was reorganized in 2017–2018, the control of the implementation of the law was partially ensured. The lack of control by the relevant body most likely led to the maintenance of the prevalence rate of exposure to flagrant messages among students who participated in the survey in 2018^30^.

### Limitations

Limitations of the study include the lack of a sufficient number of computers in the computer classes that limited the possibility to involve a greater number of students in the survey in 2015. For the ICT lessons, the classes are divided usually into at least two groups.

## CONCLUSIONS

A general increasing trend in cigarette use was observed among students from Chisinau during 2015–2018. However, despite rather strict regulations on tobacco use in Moldova, adolescents still reported a significant increase in the prevalence rate of cigarette consumption in the past 30 days. Fortunately, the frequency of anti-smoking messages has significantly increased and has been seen and read by students in shopping malls, supermarkets, cinemas, magazines, and newspapers. We highlight the importance of community preventive interventions in Moldovan schools, sustained by targeted laws, in order to reduce the frequency of smoking and associated chronic diseases.

## Data Availability

The data supporting this research are available from the authors on reasonable request.
